# Origin of the Diversity in DNA Recognition Domains in Phasevarion Associated *modA* Genes of Pathogenic *Neisseria* and *Haemophilus influenzae*

**DOI:** 10.1371/journal.pone.0032337

**Published:** 2012-03-23

**Authors:** Jayde A. Gawthorne, Scott A. Beatson, Yogitha N. Srikhanta, Kate L. Fox, Michael P. Jennings

**Affiliations:** 1 Institute for Glycomics, Griffith University Gold Coast, Queensland, Australia; 2 Australian Infectious Diseases Research Centre, School of Chemistry and Molecular Biosciences, The University of Queensland, Queensland, Australia; University of Padova, Italy

## Abstract

Phase variable restriction-modification (R-M) systems have been identified in a range of pathogenic bacteria. In some it has been demonstrated that the random switching of the *mod* (DNA methyltransferase) gene mediates the coordinated expression of multiple genes and constitutes a phasevarion (phase variable regulon). ModA of *Neisseria* and *Haemophilus influenzae* contain a highly variable, DNA recognition domain (DRD) that defines the target sequence that is modified by methylation and is used to define *modA* alleles. 18 distinct *modA* alleles have been identified in *H. influenzae* and the pathogenic *Neisseria*. To determine the origin of DRD variability, the 18 *modA* DRDs were used to search the available databases for similar sequences. Significant matches were identified between several *modA* alleles and *mod* gene from distinct bacterial species, indicating one source of the DRD variability was via horizontal gene transfer. Comparison of DRD sequences revealed significant mosaicism, indicating exchange between the *Neisseria* and *H. influenzae modA* alleles. Regions of high inter- and intra-allele similarity indicate that some *modA* alleles had undergone recombination more frequently than others, generating further diversity. Furthermore, the DRD from some *modA* alleles, such as *modA12*, have been transferred *en bloc* to replace the DRD from different *modA* alleles.

## Introduction

Restriction-modification (R-M) systems are ubiquitous in bacteria and are involved in protecting the host cell from the invasion of foreign DNA [1]. R-M systems are comprised of two components, a methyltransferase (Mod) and a restriction endonuclease (Res). Mod catalyses the methylation of host DNA at a specific nucleotide within a defined recognition sequence, allowing for the recognition of self DNA and Res catalyses the cleavage of unmethylated DNA [2]. R-M systems are classified into four groups on the basis of subunit composition, cleavage position, sequence specificity and co-factor requirements [3].

Type I R-M systems are comprised of three subunits, S, M and R, which together form a holoenzyme that performs both methylation and restriction activity. Type I systems cleave DNA at random, often far from their recognition sequences [4],[5]. Type II systems are the most common of the four with most commercially available restriction enzymes belonging to this class [3]. Type II systems consist of two independently acting enzymes for methylation and restriction, each encoded by a separate gene. Type II restriction enzymes cleave DNA at very defined positions within or close to their recognition sequences, making them valuable laboratory tools [6]. Type IV systems are extremely similar to type II systems in that methylation and restriction are catalysed by independently acting enzymes but the restriction endonuclease requires a methyl donor for successful cleavage [3].

Type III systems are complex although they only consist of two subunits, the methyltransferase and restriction endonuclease. Mod can function independently to methylate one strand of DNA within an asymmetric 5–6 bp recognition sequence [7], [8]. Res must form a complex with Mod for restriction activity as Mod contains the DNA recognition domain (DRD) [9]. Res cleaves unmethylated double-stranded DNA outside the recognition sequence, 25–27 base pairs to one side [8].

**Figure 1:**
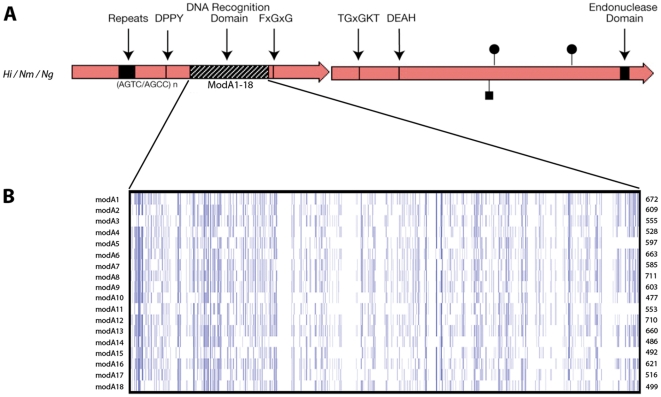
Diagrammatical representation of *modA* and *res* genes of *H. influenzae*, *N. meningitidis* and *N. gonorrhoeae*. A. The methyltransferase (*modA*) genes, and restriction endonuclease (*res*) genes, with the repeat regions that mediate phase variation. The DNA recognition domain is represented by the striped box [14]. B. The variable regions for each of the 18 *modA* alleles in the multiple sequence alignment were aligned in ClustalW and visualised with JalView using the overview feature. The nucleotides are represented as vertical bars coloured according to consensus identity (dark blue >80% identity; light blue >50% identity; white <50% identity or gap). The *mod* alleles are as follows: *mod*A1 *Hi* R3327 (126508350); *mod*A2 *Hi* 723 (126508378); *mod*A3 *Hi* R3366 (126508386); *mod*A4 *Hi* 3579 (126508396); *mod*A5 *Hi* 1268 (126508406); *mod*A6 *Hi* C1626 (126508412); *mod*A7 *Hi* R3265 (126508420); *mod*A8 *Hi* C505 (126508426); *mod*A9 *Hi* 1209 (126508430); *mod*A10 *Hi* R3157 (126508438); *mod*A11 *Nm* BZ83 (257124056); *mod*A12 *Nm* 129E (257124138); *mod*A13 *Ng* 1291 (26506021); *mod*A14 *Hi* R1527 (126508442); *mod*A15 *Hi* R3570 (126508452); *mod*A16 *Hi* ATCC9007 (126508448); *mod*A17 *Hi* ATCC9833 (126508446); *mod*A18 *Nm* NGE28 (257124039). As each *mod* allele is a different length, the number of base pairs is indicated to the right of the nucleotide alignment.

Genetic analysis of type III R-M systems has shown that the *res* gene of *H. influenzae* and the pathogenic *Neisseria* is highly conserved with DEAD box motifs commonly associated with the superfamily II helicases, see Figure 1 [10]. These are the ATP-binding motif, TGxGKT, the ATP-hydrolysis motif, DEAH, and the endonuclease motif, PD(x)_17_(D/E)xK. The *mod* gene however, contains conserved 5′ and 3′ regions that flank a highly variable, central region [10], [11]. The conserved regions of the *modA* gene encode the *S*-adenosyl-l-methioine (AdoMet) binding pocket, FxGxG, and a catalytic region, DPPY, which accommodates the target adenine in the recognition sequence after it has been flipped out of the double-helix, see Figure 1 [11], [12]. The variable region contains the DRD and dictates the sequence specificity for the methyltransferase [13].

Sequence analysis of the type III *mod* genes of pathogenic bacteria has identified simple tandem repeats that allow phase variable expression [14]. Phase variation is the reversible, high-frequency, on/off switching of gene expression and is often mediated by simple tandem repeats in the open reading frame or promoter region of genes encoding surface expressed virulence determinants, for review see Moxon *et al.* [15]. Phase variation results in the presence or absence of certain surface components and a phenotypically diverse population [16]. Methyltransferases are not surface expressed, nor are they involved in the biosynthesis of a cell surface structure, and so they represent a unique class of phase variable genes. There are several examples of phase variable type III R-M systems in host adapted bacterial pathogens, such as *Haemophilus influenzae* [17], *Neisseria gonorrhoeae* and *Neisseria meningitidis* [18], [19], *Helicobacter pylori* [20], *Pastuerella haemolytica* [21] and *Moraxella catarrhalis* [22], for review see Srikhanta *et al.* [23]. The identification of phase variable R-M systems has indicated a possible role in gene regulation through differential methylation of the genome. The *mod*A gene of *H. influenzae* [24] and both pathogenic *Neisseria* species [19] has been shown to phase vary, and to coordinate the expression of multiple genes important in pathogenesis, constituting a phase variable regulon, “phasevarion”.

The central region defines the *modA* allele as it contains the DNA recognition domain (DRD) that is responsible for DNA recognition sequence specificity. Diversity in this region has been reported [25], and further analysis on a set of 59 capsulated (typeable) and non-typeable *H. influenzae* strains defined a set of 15 distinct *modA* alleles (*modA1–10* and *modA13–17*) and three additional *modA* alleles in the pathogenic *Neisseria* (*modA11–12* and *modA18*) [14]. By definition, *mod* alleles exhibit high conservation of the DRD sequence (>95% nucleotide identity) within groups, but very little similarity between groups. To date, all reported *modA* DRD sequences have been classified as belonging to one of these 18 *mod* alleles. The *modA* alleles of *H. influenzae* and pathogenic *Neisseria* are essentially the same gene with evidence of horizontal transfer between the two species [26]. Some of the alleles are common to *H. influenzae* and *Neisseria*, such as *modA13* and *modA15*, while others are only associated with one, for example *modA11–12* are *Neisseria* specific [27]. Interestingly, most *N. meningitidis* strains have a second phase variable methyltransferase, *modB* [19] and a third, *modD*, has been recently identified in the hypervirulent *N. meningitidis* clonal complex 41/44 [28].

In the current study we undertake comprehensive bioinformatics analysis of the 18 available *modA* alleles in order to better understand the mechanisms giving rise to DRD sequence diversity.

## Materials and Methods

### Sequences

Nucleotide and amino acid sequences for the 18 *modA* alleles were obtained from GenBank. Each of the *modA* sequences had been previously described in one of three previous studies: A) 112 *modA* sequences encompassing the DRD domain, and at least 330 nt upstream and 450 nt downstream [19]; B) 22 sequences, 1188 nt to 5262 nt in length, encompassing part of *modA* 5′ and DRD up to the entire *mod/res* region [25]; and C) 54 *modA* DRD sequences, 477 nt to 711 nt in length [14]. Inter-allele comparisons were carried out with representative sequences chosen from the numerically dominant variant for each DRD allele: *modA1 Hi* R3327 (126508350); *modA2 Hi* 723 (126508378); *modA3 Hi* R3366 (126508386); *modA4 Hi* 3579 (126508396); *modA5 Hi* 1268 (126508406); *modA6 Hi* C1626 (126508412); *modA7 Hi* R3265 (126508420); *modA8 Hi* C505 (126508426); *modA9 Hi* 1209 (126508430); *modA10 Hi* R3157 (126508438); *modA11 Nm* BZ83 (257124056); *mod*A12 *Nm* 129E (257124138); *mod*A13 *Ng* 1291 (26506021); *modA14 Hi* R1527 (126508442); *modA15 Hi* R3570 (126508452); *modA16 Hi* ATCC9007 (126508448); *modA17 Hi* ATCC9833 (126508446); *modA18 Nm* NGE28 (257124039). Additionally, 77 *N. meningitidis modA12* sequences from dataset B [19], were used in an intra-allele comparative analysis.

### Sequence Analysis

Inter-allele comparisons were carried out using all-versus-all BLASTn and BLASTp comparisons of representative *mod* DRD alleles using unfiltered stand-alone BLAST (version 2.2.18). To identify homologous DRD sequences, the amino acid sequence for each representative *modA* DRD allele was used as the query sequence for tBLASTn searches of the nucleotide databases at NCBI, including the whole-genome-shotgun and environmental databases. *Mod* and *res* loci from complete and unfinished genomes were identified by using the *H. influenzae* KW20 *modA1* 4.6 kb locus to query the GenBank nucleotide database and Whole Genome Shotgun database, respectively. Matches were further analysed using Artemis Comparison Tool [29] to retrieve the entire *mod* and *res* sequences and determine if either were pseudogenes. The *modA* alleles for 38 genome-derived *mod* sequences were determined by unfiltered stand-alone BLASTn against a custom database consisting of representative *modA* DRD allele nucleotide sequences. Unless otherwise stated, all BLAST searches were carried out with default parameters. Amino acid and nucleotide sequences were aligned using ClustalX 2.0.11 with default parameters. Alignments were viewed and edited using Jalview 2.4.0.b2 [30]. Sequence alignments were analysed for evidence of recombination using PhiPack [31]. Phylogenetic analyses were carried out using the PHYLIP package [32]. Sequence alignments used in Figures 1, 4 and 6 are provided in Data S1.

## Results

### Detailed analysis of the diversity of the DRD domain that defines *modA* alleles

Representatives of all 18 *modA* alleles were compared by multiple sequence alignment. Figure 1 illustrates the diversity seen throughout the DRD of the *modA* alleles at the nucleotide level with respect to the nucleotide sequence and length of the DRD. The closest *mod*A DRD domains share 38% amino acid identity, whereas the most distant share 29%. To probe the origin of DRD diversity and the relative contribution of intra- and inter-species recombination mechanisms we undertook a detailed analysis of all available *modA* alleles.

### Source of *modA* Diversity: Inter-species recombination

Our previous work [14] has identified homology between particular *modA* DRDs and DNA methyltransferases from unrelated bacterial species suggesting acquisition of new DRDs *en-bloc* via horizontal gene transfer. To further explore the source of extant *modA* alleles, BLAST searches using all available *modA* DRD regions were carried out. We used the amino acid sequences for the DRD of each *modA* allele to undertake tBLASTn searches of NCBI nucleotide databases to identify likely source genomes. Ignoring self-matches, all matches with >50% amino acid identity (*E*-value <0.001) identified in the WGS division of Genbank are outlined in table 1. Notably, some of the most significant matches were to other upper respiratory tract bacterial species, for example *Legionella pneumophila* and *Moraxella catarrhalis*. As previously stated, the amino acid sequence identity of the DRD between known *modA* alleles (i.e. ModA1–ModA18) is >29%, therefore the identification of matches >50% between the *modA* DRD and non-related bacterial species required further investigation. In all cases the match was with a predicted adenine specific type III R-M system methyltransferase. Matches between *modA4* and *Helicobacter sp.* [20] and *modA5* and *Moraxella catarrhalis* [22] are with methyltransferases that are predicted phase variable, however the repeat regions mediating phase variation are different.

**Table 1:** Significant matches identified with tBLASTn.

*modA* Allele^a^	Match^b^	GenBank Accession^c^	% Id^d^	% Sim^d^	Length of Match^e^
*modA1*	*Fusobacterium* sp. 3_1_33 cont1.59	ACQE01000059.1	52	72	228
*modA2*	*Enhydrobacter aerosaccus* SK60 contig00039	ACYI01000007.1	72	83	210
*modA4*	*Helicobacter acinonychis* str. Sheeba	AM260522	57	72	176
	*Helicobacter pylori* G27	CP001173	56	71	176
	*Helicobacter pylori* P12	CP001217	56	72	176
	*Helicobacter pylori* Shi470	CP001072	56	71	176
	*Helicobacter pylori* 26695	AE000511	55	70	176
*modA5*	*Haemophilus parasuis* SH0165	CP001321	69	81	199
	*Fusobacterium* sp. 4_1_13 cont1.41	ACDE01000041.1	68	79	199
	*Clostridium perfringens* CPE str. F4969 gcontig_1106202596928	ABDX01000002.1	57	76	200
	*Stenotrophomonas* sp. SKA14 ctg_1108481805199	ACDV01000027.1	50	69	200
	*Moraxella catarrhalis*	AY049057.1	73	87	200
*modA6*	*Helicobacter pylori* B128 contig00164	ABSY01000012.1	66	80	220
*modA7*	*Lactobacillus jensenii* 269-3 contig00058	ACOY01000053.1	53	69	196
	*Lactobacillus jensenii* 1153 cont1.15	ABWG01000015.1	53	69	196
*modA15*	*Legionella pneumophila* str. Paris	CR628336	69	83	164

a As dictated by the *mod* variable region.

b Significant matches from the microbial genome, non-redundant, or environmental databases.

c GenBank accession number for each significant match.

d Identity and similarity values correspond to the entire *mod* variable region.

e Length of match in amino acids DRD lengths: *modA4* 176aa, *modA5* 199aa, *modA6* 221aa, *modA7* 196aa, *modA15* 164aa.

**Figure 2:**
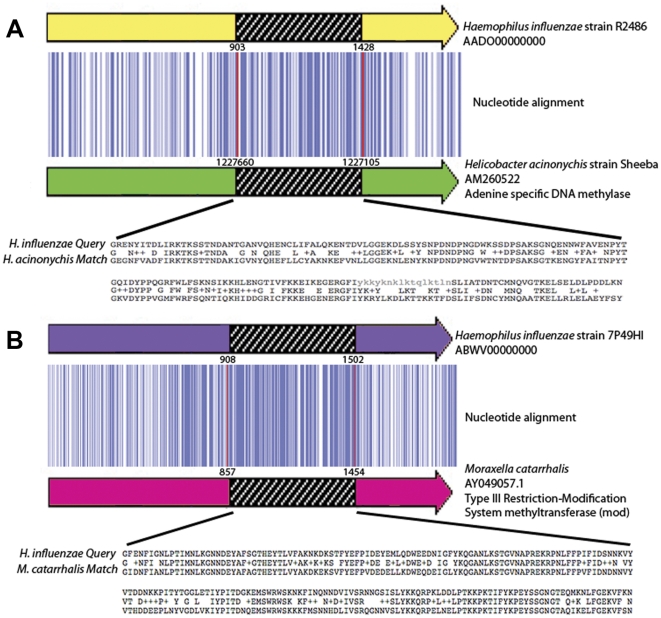
Diagrammatical representation of significant matches. A. Diagrammatical representation of the *H. influenzae* and *Helicobacter acinonychis mod* alignment. The tBLASTn local alignment identified between the DNA recognition domain of *mod*A4 and *Helicobacter acinonychis* was further extended to include the entire *modA* gene of *H. influenzae*. A nucleotide alignment was completed to include the regions flanking the DRD. The yellow arrow represents entire *modA* gene and the green arrow the match in *H. acinonychis*. B. Diagrammatical representation of the *H. influenzae* and *Moraxella catarrhalis mod* alignment. The tBLASTn local alignment identified between the DNA recognition domain of *mod*A5 and *Moraxella catarrhalis* was further extended to include the entire *modA* gene of *H. influenzae*. A nucleotide alignment was completed to include the regions flanking the DRD. The purple arrow represents the entire *modA* gene in *H. influenzae* and the pink arrow the match in *M. catarrhalis*. The nucleotides are represented as vertical blue bars (dark blue >80% identity; light blue >50% identity; white >50% identity or gap). Strain and accession numbers that define the matches are shown to the left. The position of nucleotide that the match occurs is indicated above the coloured arrows and beside the amino acid alignment.

The local alignments identified by BLAST were extended using the corresponding complete sequences to determine the actual length of the match and determine if the alignment extended beyond the boundaries of the *modA* DRD. The matches between the *modA4* and *Helicobacter acinonychis* Hac_1417 and *modA5* and *Moraxella catarrhalis* McaRII are shown in figure 2. In both cases it appears that the nucleotide identity extends beyond the DRD boundaries, which are highlighted with vertical red lines (Figure 2), suggesting that the conserved sequence flanking the variable region could have provided a long homologous area for recombination to occur.

### Source of *modA* Diversity: Inter-allele recombination

**Figure 3:**
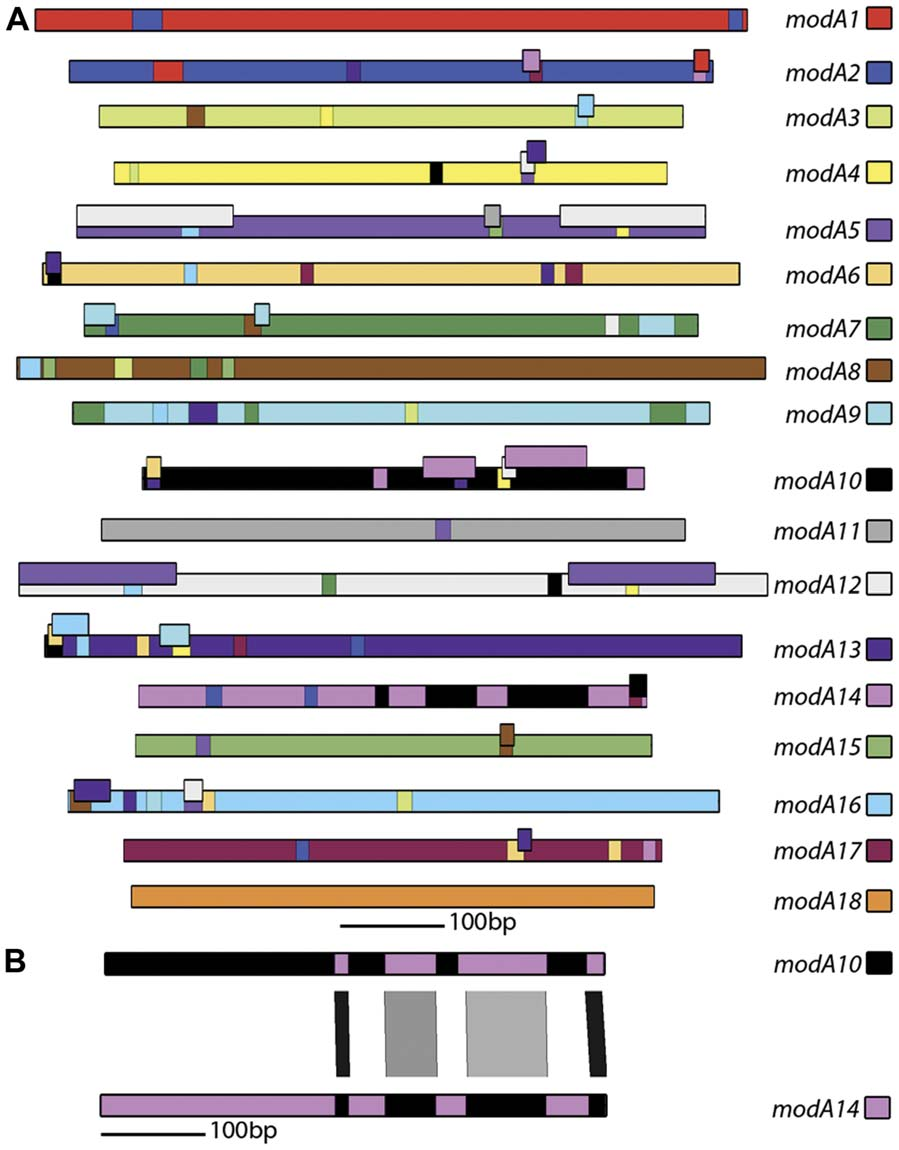
Diagrammatical representation of the 18 *modA* alleles of *H. influenzae* and pathogenic *Neisseria*. A. The 18 *modA* DNA recognition domains are shown as coloured lines and the colour scheme established in Fox *et al.* (14) was used here for each representative allele: 1, *H. influenzae* R3327; 2, *H. influenzae* 723; 3, *H. influenzae* R3366; 4, *H. influenzae* R3579; 5, *H. influenzae* 1268; 6, *H. influenzae* C1626; 7, *H. influenzae* R3265; 8, *H. influenzae* C505; 9, *H. influenzae* 1209; 10, *H. influenzae* R3157; 11, *N. meningitidis* BZ83; 12, *N. gonorrhoeae* 01DO64; 13, *H. influenzae* R3023; 14, *H. influenzae* R1527; 15, *H. influenzae* R3570; 16, *H. influenzae* ATCC9007; 17, *H. influenzae* ATCC9833; 18, *N. meningitidis* NGE28. Each *modA* DNA recognition domain has been assigned a unique colour to permit visual identification of possible recombination events between different *modA* DNA recognition domains. BLASTn matches longer than 13 nucleotides and >80% identity between the 18 *mod* alleles were mapped onto the corresponding allele in the appropriate colour. Table S1 contains the nucleotide coordinates for each BLASTn match. B. Diagrammatical representation of the tBLASTn match between *modA10* and *modA14*. Horizontal bars represent the *modA* alleles 10 and 14 described in panel A, and are coloured according to the same scheme. The vertical bars between the coloured lines indicate the nucleotide match according to BLAST, ranging from light grey (>80% nt identity) to black (100% nt identity).

To examine the contribution of recombination between the DRDs of *modA* genes within the same species to overall *modA* allele diversity, we undertook all-versus-all BLASTn searches using the 18 representative *modA* sequences. Matches between the alleles identified with a length greater than 13 nucleotides were mapped onto the corresponding allele and coloured according to the scheme established in Fox *et al.* [14]. The number of reciprocal exchanges identified was a clear indication that the *modA* alleles have recombined in the past. Most interestingly, new relationships between the *modA* alleles were identified, and some alleles were found to have undergone recombination much more readily than others, indicating possible selection. Figure 3B shows the close relationship identified between *modA10* and *modA14*. The DRDs show evidence of recombination with long stretches of nucleotide identity (>80%) between the two alleles. Sequences within the same *modA* allele share very high identity over the DRD region (>95%) suggesting that recombination is not presently contributing to the diversity. Indeed, the high conservation suggests that the DRD is moving as a unit. To investigate this further we undertook an analysis of a particular *modA* allele where a large number of sequences are available.

### Source of *modA* Diversity: Intra-allele recombination

**Figure 4:**
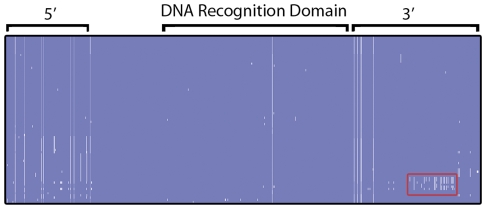
Alignment profile for 77 *N. meningitidis modA12* alleles and flanking regions. The DRD variable domain, 330 nt upstream and 450 nt downstream were aligned using ClustalW and visualized using Jalview. The nucleotides are represented as vertical bars coloured according to consensus identity (dark blue >90% identity; light blue >50% identity; white <50% identity). Highlighted in red is a block of sequence indicative of recombination, where some sequences are identical to equivalent regions of *H. influenzae* PittEE, *modA6*.

Despite evidence for recombination between *modA* alleles, it is apparent from previous studies and our own analysis that the DRD domains are highly conserved amongst members of the same *modA* allele [14], [19]. To further examine how diversity arises within *modA* alleles nucleotide alignments were completed with 77 available sequences for the *N. meningitidis modA12* DRD alleles and their flanking regions [19] (Figure 4). The DRD was remarkably well conserved across all the analysed *modA12* sequences (0.2% nt diversity, across 895 nt of sequence) (Table 2). In contrast, the intra-allele variability was found in the regions flanking the DRD domain (Table 2). Throughout the entire alignment, the majority of nucleotide substitutions gave rise to non-synonymous substitutions suggesting that some codons may be subject to positive selection, as previously described [25]. Interestingly, the alignments showed discrete blocks of sequence divergence, mostly confined to the 3′ region of the *modA* gene, that were suggestive of recombination, rather than selection (Figure 4). A closer examination of these sequences indicate that these blocks match the nucleotide sequence of other *modA* alleles; for example, a short segment in the 3′ end of some *modA12* alleles was observed to be a perfect match to the 3′ end of the *modA6* alleles (Figure 4). To formally test for recombination we used PhiTest [31] which implements a pair-wise homoplasy test. There was good evidence for recombination in the 3′ region, but not the DRD or 5′ region of the *modA12* alleles (Figure 4), consistent with the *modA6* observation. To examine if similar evolutionary processes were shaping the diversity of other *modA* groups we wanted to take advantage of the availability of entire *modA* loci as part of complete and unfinished genome projects.

**Table 2:** Sequence analysis of *N. meningitidis modA12* genes.

		GC-content	Diversity	Phi	Recomb?	Ns sites^a^
*modA12*_5prime	330 nt	40%	2.40%	3.70E-01	N	6
*modA12*_DRD	895 nt	28%	0.20%	9.38E-01	N	4
*modA12*_3prime	450 nt	40%	1.50%	1.89E-29	Y	10

a Ns = Non-synonymous sites with more than 2 sequences contain a Non-synonymous substitution.

### DRD acquisitions *en bloc* between pathogenic *Neisseria*

**Figure 5:**
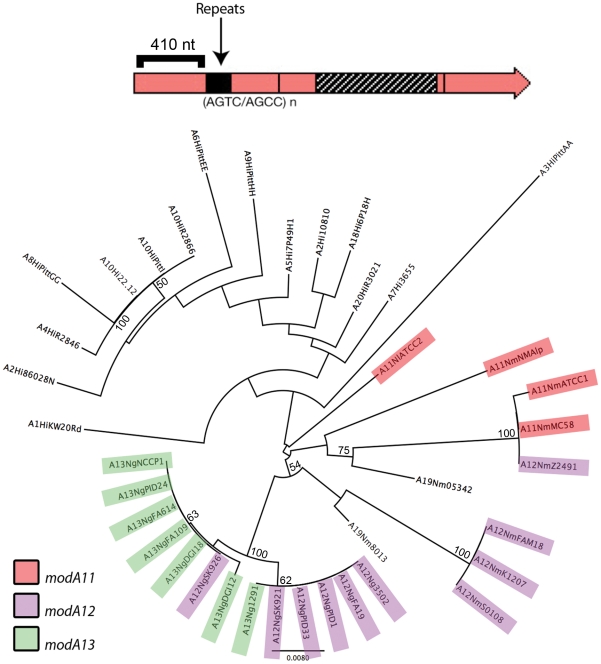
An unrooted phylogram shows the relationship between 5′ regions of *modA* genes (ATG to repeat, 410 nt). Phylogram of the 410 nt *modA* 5′ nucleotide sequence produced using the Neighbor-joining algorithm, as implemented in PHYLIP [32]. Taxa that do not cluster according to their cognate *mod* allele are highlighted: *N. meningitidis* and *N. gonorrhoeae modA11* (red), *modA12* (purple) and *modA13* (green). Bootstrap values from 1000 replications are shown as percentages for nodes with more than 50% support. Scale bar shows distances in nucleotide substitutions per site.

The sequence conservation between the DRD of each *modA* allele is >95% at the nucleotide level (Table S2). To determine if the conserved regions flanking each DRD contained evidence of previous recombination, a phylogenetic analysis was undertaken using the available *H. influenzae*, *N. meningitidis* and *N. gonorrhoeae* genomes. BLASTn searches with the *H. influenzae* Rd 4.6 kb *modA1* region identified matches in 38 complete or unfinished genomes. Subsequent analysis enabled the identification of *mod* and *res* genes in most cases (several genomes contained frame-shifts in *mod* or *res*, or suspected repeat-tract “OFF” variants, Table S3). For each sequence, the 5′ region (between the ATG start codon and the beginning of the tetranucleotide repeats) were retrieved and aligned. A phylogram of this alignment shows that the 5′ regions of *modA* tend to cluster with their species of origin, rather than with their cognate *modA* DRD allele (Figure 5). Identical 5′ *modA* regions are found to be associated with *modA11* and *modA12* alleles in *N. meningitidis* MC58 and Z2491, respectively; whereas the 5′ region of *modA12* from several *N. gonorrhoeae* strains are identical to that found in a *modA13* allele from *N. gonorrhoeae* 12291 (Figure 5). A similar observation can be made for the 5′ regions of *modA4* and *modA10* alleles from *H. influenzae*, which are near-identical. This result is consistent with other findings e.g. *modA4* from *H. influenzae* and *N. gonorrhoeae*. Therefore, the *modA* alleles are in a constant flux, retaining a high degree of conservation within DRD domains that are able to recombine into different *modA* “back-bones” and overwrite the existing allele by homologous replacement.

### Analysis of *modA* Alleles in Available Genome Sequences

**Figure 6:**
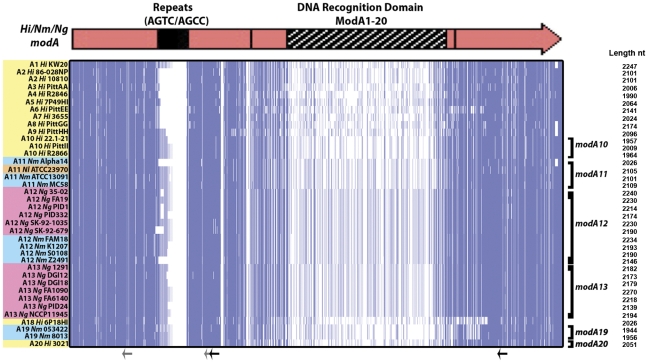
Multiple nucleotide sequence alignment of complete *modA* sequence from available genome sequences. The *modA* genes from *H. influenzae* (yellow), *N. gonorrhoeae* (pink), *N. meningitidis* (blue) and *N. lactamica* (orange) isolates were aligned. Nucleotides are represented as vertical bars (dark blue >80% identity; light blue >50% identity; white <50% identity or gap). Strain names are indicated on the left of the nucleotide alignment and major *modA* alleles represented here are indicated on the right. Arrows indicate the position of *Neisserial* uptake sequence 5′-GCCGTCTGAA-3′ (grey are imperfect matches, 8/10 in majority of strains; black arrows are perfect matches). Sequences were aligned in ClustalW and displayed using JalView (jalview.org).

A multiple nucleotide alignment of all *modA* gene sequences identified in table S3 was generated (Figure 6). Although several new strains containing the *modA*12 and *modA13* alleles were identified, no representatives of *modA14–17* were found in the new genome sequence projects. Three *modA* genes were found to have central regions with no significant matches at the nucleotide level to known *modA* DRD alleles. We propose that the DRD regions define new *modA* alleles called *modA19* (*N. meningitidis* 053422 and *N. meningitidis 8013*) and *modA20* (*H. influenzae* R3021), respectively.

As previously shown (Figure 4), the data in figure 6 indicates recombination events of large fragments that encompass the entire DRD region; this is particularly obvious in the 3′ regions of the *modA6*, *modA18* and *modA19* alleles. In the case of *modA19*, the high conservation within the DRD, coupled with the relatively non-conserved sequence at either end suggests independent homologous recombination events of the entire DRD region from an unknown source into different *modA* alleles. This suggests that the same DRD allele should be found within *modA* genes with less identity in their flanking regions. The identification of *Neisserial* specific uptake sequences (nUSS), 5′-GCCGTCTGAA-3′, flanking the variable domain in all the aligned genome sequences in this study and *H. influenzae* specific uptake sequences (hUSS) in a previous study [26], both from pathogenic *Neisseria* and *H. influenzae*, indicates that the DRD would be preferentially transformed into these species following cell death, lysis, then natural transformation of random genomic fragments. nUSS and hUSS represent a well characterised feature used to enhance transformation efficiency [33] and constitute a predicted 1% of the genome [34]. The association of the nUSS with the *modA* DRD is consistent with the hypothesis that the variable domain is frequently transferred between *H. influenzae*, *N. gonorrhoeae* and *N. meningitidis*. The comparison of the relative GC content is also a good indicator that a specific region of DNA originates outside its current organism. The calculated GC content of the DRD of the analysed *modA12* sequences is significantly lower than either the 5′ or 3′ regions of the *modA* gene, see table 2, providing further evidence of acquisition via horizontal gene transfer.

## Discussion

Investigating the origins of the distinct *modA* phasevarions in *Haemophilus influenzae* and the pathogenic *Neisseria* forms the basis of this study. The *modA* alleles in *Haemophilus* and pathogenic *Neisseria* contain conserved 5′ and 3′ coding regions, which flank a highly variable central region. The origins of the diversity of the central, variable region, the DRD, have been previously investigated by Bayliss *et al.* (25), although this analysis only identified 13 *modA* alleles as fewer strains were examined. A further 5 distinct *modA* alleles have been determined through sequencing of *modA* genes in *H. influenzae* [14] and *N. gonorrhoeae* and *N. meningitidis* [19], therefore we undertook a new analysis with all known 18 *modA* alleles and took advantage of the increase in publicly available bacterial genome sequences in recent years. Our sequence analysis of the publicly available genomes led to the discovery of an additional 2 *modA* alleles, which we designate *modA19* and *modA20*.

The diversity seen between the 20 *modA* alleles has been generated by both small and large scale recombination between species and strains at various evolutionary time-points. The first source of novel DRD sequences that we investigated were other bacterial species. All available bacterial genomic sequences located in the NCBI databases were searched using tBLASTn to identify the initial matches between other bacterial species and the *modA* DRD. In all cases the most significant matches were with putative adenine-specific type III R-M methyltransferases. There have been over 500 putative type III methyltransferases identified but only a small number of these have been completely characterised [35] and so there is little experimental data available for the *modA* DRD matches. However, the *modA4* matches with *H. pylori* are homologs of the methyltransferase, JHP1296 or *modH1*, which has been shown to phase vary [20]. The switching of expression of the *H. pylori* methyltransferase has been demonstrated with phase-variable *lacZ* expression [20], indicating that this system may possibly be shifting from its traditional role in host genome protection to one of gene regulation. Additionally, *modA5* matched a putative phase variable methyltransferase in *M. catarrhalis* [22]. The *M. catarrhalis mod* was predicted to phase vary based on the presence of tetranucleotide repeats in the open reading frame of a type III R-M system [22]. The identification of phase variable methyltransferases in other host-adapted pathogens indicated that this system of gene regulation, the phasevarion, could be important for host adaption and immune system evasion.

Early in the evolution of *modA*, diversity was generated by the transfer of short segments of the DRD between different *H. influenzae* and *Neisseria modA* genes. There are known elements associated with the *modA* genes that have been established as allowing the transfer to occur, namely *H. influenzae* and *Neisserial* specific uptake sequences and remnants of an integrase [26]. Our analysis revealed that there were a number of segments found within the DRD that displayed a high sequence similarity to other *modA* alleles, and it is proposed that these segments have been transferred between the different *modA* alleles. It is hypothesised that recombination between different *modA* DRDs affects the sequence specificity recognised by the *modA* allele and therefore can play a role in the fitness of the organism to an evolving host environment by introducing new regulatory patterns.

Within the same *modA* allele, strains were found to differ in ≤5% of their nucleotide sequence [14], [25]. The *modA12* allele was chosen for an in depth analysis of intra-allele variability as it was the most highly represented group in available *modA* sequences and has recently been reported as a functional phasevarion [19]. The majority of variation was found outside of the established DRD boundaries suggesting strong selective pressure to maintain the *modA12* DRD sequence in these strains. Indeed, the *modA12* DRD sequences examined here exhibited only 0.2% diversity within the DRD itself. Bayliss *et al.* [25] reported that the presence of stretches of homologous sequence between the DRDs of *modA* alleles indicated that it was a dynamic area that was in a constant flux. Notably, that analysis used only a handful of representative sequences. In contrast, recent recombination between the DRDs of different *modA* alleles to generate diversity does not hold with our analysis of 77 *modA12* sequences. Instead the *modA12* sequences show evidence of other *modA* alleles in their 5′ and 3′ regions, indicating that the entire *modA12* DRD had “over written” a previous allele. We propose that the intra-allele sequence shuffling occurred early in the acquisition of the DRD by either *H. influenzae* or *Neisseria* and that there is a strong selection to maintain the current sequence of the *modA12* DRD.

In summary this study has characterised the diversity between the 20 *modA* alleles and examined origins of the significant diversity seen in the *modA* DRD domain. We have shown that several DRD regions of *modA* alleles found in pathogenic *Neisseria* and *H. influenzae* were acquired *en-bloc* from type III R-M systems from unrelated bacterial species. Subsequent diversity is generated by recombination between *modA* alleles via frequent horizontal transfer between and within these two species; in some cases this has involved the transfer of the entire DRD region. We find remarkable conservation within any given *modA* DRD, even when they are found in different genetic contexts. Accordingly, *modA* alleles should be defined only by the DRD region itself, and exclude the flanking 5′ and 3′ regions that often bare the scars of multiple recombination events. Our previous work on the pathogenic *Neisseria* has shown that distinct *modA* alleles recognised distinct DNA targets sites and thus regulate distinct regulons of genes with key roles in model systems of disease [19]. Amid the high frequency exchange and recombination driving the generation of this diverse set of *modA* alleles we observed selection for maintenance the *modA12* allele in *N. meningitidis*. This indicates a strong selective pressure and benefit conferred by presence of the *modA12* phasevarion in these *N. meningitidis* strains. The *modA12* allele is the *modA* allele most frequently observed in *N. meningitidis* disease isolates (78.5%; [19]). The wide dissemination of the *modA12* allele suggests that the ability to alter *modA* sequence specificity and the concomitant phasevarion in a single step may be key to the survival of some pathogenic strains. The original source of DRDs sequence diversity in restriction systems was undoubtedly generated by phage resistance as a dominant selective pressure [36]. However, in the case of *modA* alleles constituting phasevarion regulatory systems in *H. influenzae* [14], the pathogenic *Neisseria* [19], and also *modH* phasevarions of *H. pylori* [37], the *mod* genes are frequently associated with an inactive *res* (restriction) component of the Type III system, indicating selection for particular *mod* alleles observed in these studies is likely to be driven by the advantage conferred by the gene regulation phenotype, not by a restriction related function.

## Supporting Information

Table S1 **Significant BLASTn matches between** ***modA*** **DNA recognition domains of** ***modA*** **alleles in** ***H. influenzae*** **and the pathogenic** ***Neisseria*** **.**
(DOCX)

Table S2 **Within-allele diversity of DRD sequences.**
(DOC)

Table S3 ***modA*** **alleles identified from available genomes.**
(DOCX)

Data S1 (ZIP)
