# Supplementary material for: Origin of the Diversity in DNA Recognition Domains in Phasevarion Associated modA Genes of Pathogenic Neisseria and Haemophilus influenzae
Source: PLoS One. 2012 Mar 23;7(3):e32337. doi: 10.1371/journal.pone.0032337 (PMC3311624; doi:10.1371/journal.pone.0032337)
Supplement: Table S1 — Significant BLASTn matches between modA DNA recognition domains of modA alleles in H. influenzae and the pathogenic Neisseria . (DOCX) [file pone.0032337.s001.docx]

**Table S1. Significant BLASTn matches between *modA* DNA recognition domains of *modA* alleles in *H. influenzae* and the pathogenic *Neisseria***

| ***modA* Allele^a^** | ***modA* match^a^** | **Match Accession** | **5’ Hit^b^** | **3’ Hit^b^** |
| --- | --- | --- | --- | --- |
| 1 | 2 | 126508378 | 92 | 120 |
| 1 | 2 | 126508378 | 655 | 668 |
| 2 | 1 | 126508350 | 80 | 108 |
| 2 | 13 | 126508450 | 263 | 276 |
| 2 | 14 | 126508442 | 430 | 445 |
| 2 | 17 | 126508446 | 436 | 448 |
| 2 | 14 | 126508442 | 591 | 603 |
| 2 | 1 | 126508350 | 592 | 605 |
| 3 | 8 | 126508426 | 84 | 101 |
| 3 | 4 | 126508396 | 211 | 223 |
| 3 | 9 | 126508430 | 453 | 465 |
| 3 | 16 | 126508448 | 456 | 470 |
| 4 | 3 | 126508386 | 16 | 28 |
| 4 | 10 | 126508438 | 302 | 314 |
| 4 | 5 | 126508406 | 389 | 401 |
| 4 | 12 | 257812140 | 389 | 401 |
| 4 | 13 | 126508450 | 395 | 412 |
| 5 | 12 | 257812140 | 1 | 149 |
| 5 | 16 | 126508448 | 100 | 117 |
| 5 | 11 | 257124056 | 388 | 402 |
| 5 | 15 | 126508452 | 392 | 405 |
| 5 | 12 | 257812140 | 460 | 597 |
| 5 | 4 | 126508396 | 513 | 525 |
| 6 | 13 | 126508450 | 4 | 17 |
| 6 | 10 | 126508438 | 5 | 18 |
| 6 | 16 | 126508448 | 135 | 147 |
| 6 | 17 | 126508446 | 246 | 258 |
| 6 | 13 | 126508450 | 475 | 487 |
| 6 | 17 | 126508446 | 498 | 514 |
| 7 | 9 | 126508430 | 1 | 30 |
| 7 | 2 | 126508378 | 21 | 33 |
| 7 | 8 | 126508426 | 153 | 169 |
| 7 | 9 | 126508430 | 163 | 176 |
| 7 | 12 | 257812140 | 497 | 510 |
| 7 | 9 | 126508430 | 529 | 563 |
| 8 | 16 | 126508448 | 3 | 23 |
| 8 | 15 | 126508452 | 25 | 37 |
| 8 | 3 | 126508386 | 93 | 110 |
| 8 | 7 | 126508420 | 165 | 181 |
| 8 | 15 | 126508452 | 195 | 207 |
| 9 | 7 | 126508420 | 1 | 30 |
| 9 | 16 | 126508448 | 76 | 90 |
| 9 | 13 | 126508450 | 110 | 137 |
| 9 | 7 | 126508420 | 163 | 176 |
| 9 | 3 | 126508386 | 315 | 327 |
| 9 | 7 | 126508420 | 547 | 581 |
| 10 | 13 | 126508450 | 5 | 17 |
| 10 | 6 | 126508412 | 5 | 18 |
| 10 | 14 | 126508442 | 220 | 233 |
| 10 | 14 | 126508442 | 268 | 317 |
| 10 | 13 | 126508450 | 297 | 309 |
| 10 | 4 | 126508396 | 338 | 350 |
| 10 | 12 | 257812140 | 343 | 356 |
| 10 | 14 | 126508442 | 346 | 423 |
| 10 | 14 | 126508442 | 461 | 477 |
| 11 | 5 | 126508406 | 317 | 331 |
| 12 | 5 | 126508406 | 1 | 149 |
| 12 | 16 | 126508448 | 100 | 117 |
| 12 | 7 | 126508420 | 288 | 301 |
| 12 | 10 | 126508438 | 502 | 515 |
| 12 | 5 | 126508406 | 523 | 660 |
| 12 | 4 | 126508396 | 576 | 588 |
| 13 | 10 | 126508438 | 1 | 13 |
| 13 | 6 | 126508412 | 4 | 17 |
| 13 | 16 | 126508448 | 7 | 41 |
| 13 | 9 | 126508430 | 110 | 137 |
| 13 | 6 | 126508412 | 87 | 99 |
| 13 | 16 | 126508448 | 30 | 42 |
| 13 | 4 | 126508396 | 121 | 138 |
| 13 | 17 | 126508446 | 179 | 191 |
| 13 | 2 | 126508378 | 290 | 303 |
| 14 | 2 | 126508378 | 65 | 80 |
| 14 | 2 | 126508378 | 159 | 171 |
| 14 | 10 | 126508438 | 226 | 239 |
| 14 | 10 | 126508438 | 274 | 323 |
| 14 | 10 | 126508438 | 352 | 429 |
| 14 | 17 | 126508446 | 468 | 480 |
| 14 | 10 | 126508438 | 470 | 486 |
| 15 | 5 | 126508406 | 59 | 72 |
| 15 | 8 | 126508426 | 348 | 360 |
| 15 | 8 | 126508426 | 349 | 361 |
| 16 | 8 | 126508426 | 3 | 23 |
| 16 | 13 | 126508450 | 7 | 41 |
| 16 | 13 | 126508450 | 54 | 66 |
| 16 | 9 | 126508430 | 76 | 90 |
| 16 | 5 | 126508406 | 112 | 129 |
| 16 | 12 | 257812140 | 112 | 129 |
| 16 | 6 | 126508412 | 129 | 141 |
| 16 | 3 | 126508386 | 315 | 329 |
| 17 | 2 | 126508378 | 166 | 178 |
| 17 | 6 | 126508412 | 368 | 384 |
| 17 | 13 | 126508450 | 378 | 390 |
| 17 | 6 | 126508412 | 465 | 477 |
| 17 | 14 | 126508442 | 498 | 510 |

^a^*modA* allele defined in figure 3. ^b^Nucleotide coordinate for the *modA* DNA recognition domain.
