# Supplementary material for: Origin of the Diversity in DNA Recognition Domains in Phasevarion Associated modA Genes of Pathogenic Neisseria and Haemophilus influenzae
Source: PLoS One. 2012 Mar 23;7(3):e32337. doi: 10.1371/journal.pone.0032337 (PMC3311624; doi:10.1371/journal.pone.0032337)
Supplement: Table S2 — Within-allele diversity of DRD sequences. (DOC) [file pone.0032337.s002.doc]

Table S2. Within-allele diversity of DRD sequences.

| **Mod allele** | **Total sequences** | **Species Distribution^1^** | **Length^2^** | **Nucleotide Identity^3^** |
| --- | --- | --- | --- | --- |
| A1 | 4 | 4Hi | 672 | 99.9-100% |
| A2 | 15 | 15Hi | 609 | 98.8-100% |
| A3 | 5 | 5Hi | 555 | 100% |
| A4 | 7 | 6Hi,1Nm | 528 | 98.7-100% |
| A5 | 6 | 6Hi | 597 | 99.8-100% |
| A6 | 5 | 4Hi | 663 | 99.8-100% |
| A7 | 6 | 5Hi | 585 | 99.7-100% |
| A8 | 4 | 4Hi | 711 | 100% |
| A9 | 4 | 4Hi | 603 | 99.8-100% |
| A10 | 7 | 7Hi | 477 | 100% |
| A11 | 21 | 20Nm,1Nl | 553 | 99.5-100% |
| A12 | 89 | 7Ng,81Nm | 710 | 99-100% |
| A13 | 8 | 7Ng,1Hi | 660 | 98.5-100% |
| A14 | 3 | 3Hi | 486 | 99.6-100% |
| A15 | 3 | 2Nm,1Hi | 492 | 97.4-100% |
| A16 | 1 | 1Hi | 621 | 100% |
| A17 | 1 | 1Hi | 516 | 100% |
| A18 | 2 | 1Nm,1Hi | 499 | 100% |

^1^ Number of representative from each species: Hi (*H. influenzae*); Nm (*N. meningitidis*); Nl (*N. lactamica*); Ng (*N. gonorrhoeae*)

^2^ Length in nucleotides of DRD sequence

^3^ Range of nucleotide identity within *mod* allele
