# Supplementary material for: Origin of the Diversity in DNA Recognition Domains in Phasevarion Associated modA Genes of Pathogenic Neisseria and Haemophilus influenzae
Source: PLoS One. 2012 Mar 23;7(3):e32337. doi: 10.1371/journal.pone.0032337 (PMC3311624; doi:10.1371/journal.pone.0032337)
Supplement: Table S3 — modA alleles identified from available genomes. (DOCX) [file pone.0032337.s003.docx]

Table S3 *modA* alleles identified from available genomes

| ***modA* Allele** | **Organism** | **Strain Id** | **Accession** | **Strand** | **Start** | **End** | ***modA* Locus Tag** |
| --- | --- | --- | --- | --- | --- | --- | --- |
| A1 | *H. influenzae* | Rd KW20 | [L42023](http://www.ncbi.nlm.nih.gov/nuccore/L42023) | - | 1124048 | 1121801 | HI_1056, HI_1055 |
| A2 | *H. influenzae* | 86-028NP | CP000057 | - | 1162380 | 1160279 | NTHI1217 |
| A2 | *H. influenzae* | 10810 | FQ312006 | - | 1254051 | 1251950 | HIB_12170, HIB_12160 |
| A3 | *H. influenzae* | PittAA | [AAZG00000000](http://www.ncbi.nlm.nih.gov/nuccore/AAZG00000000) | - | 191330 | 189324 | ctg64 |
| A4 | *H. influenzae* | R2846 | CP002276 | + | 1340963 | 1342953 | R2846_1278 |
| A5 | *H. influenzae* | 7P49H1 | [ABWV00000000](http://www.ncbi.nlm.nih.gov/nuccore/ABWV00000000) | + | 331929 | 329865 | ctg21 |
| A6 | *H. influenzae* | PittEE | [CP000671](http://www.ncbi.nlm.nih.gov/nuccore/CP000671) | + | 1343865 | 1346006 | CGSHiEE_06790 |
| A7 | *H. influenzae* | 3655 | [AAZF00000000](http://www.ncbi.nlm.nih.gov/nuccore/AAZF00000000) | - | 343744 | 341720 | ctg84 |
| A8 | *H. influenzae* | PittGG | [CP000672](http://www.ncbi.nlm.nih.gov/nuccore/CP000672) | - | 1653322 | 1651148 | CGSHiGG_08920 |
| A9 | *H. influenzae* | PittHH | [AAZH00000000](http://www.ncbi.nlm.nih.gov/nuccore/AAZH00000000) | + | 81886 | 83982 | ctg65 |
| A10 | *H. influenzae* | 22.1-21 | AAZD01000001 | + | 249508 | 251465 | ctg19 |
| A10 | *H. influenzae* | PittII | AAZI01000001 | + | 41966 | 43975 | ctg274 |
| A10 | *H. influenzae* | R2866 | CP002277 | + | 1368660 | 1370624 | R2866_1343 |
| A11 | *N. meningitidis* | Alpha 14 | [AM889136](http://www.ncbi.nlm.nih.gov/nuccore/AM889136) | - | 1266414 | 1268440 | NMO_1215 |
| A11 | *N. lactamica* | ATCC 23970 | ACEQ0200000 | - | 32044 | 29939 | Cont4.1 |
| A11 | *N. meningitidis* | ATCC 23970 | AEEF01000046 | - | 28803 | 26702 | HMPREF0602_0727, HMPREF0602_0726 |
| A11 | *N. meningitidis* | MC58 | [AE002098](http://www.ncbi.nlm.nih.gov/nuccore/AE002098) | + | 1399877 | 1401986 | NMB1375 |
| A12 | *N. gonorrhoeae* | 35-02 | ABZG01000070 | + | 6713 | 32053 | cont1.70 |
| A12 | *N. gonorrhoeae* | FA19 | ABZJ01000058 | - | 13311 | 11081 | cont1.58 |
| A12 | *N. gonorrhoeae* | PID1 | ABZM01000074 | + | 29839 | 32053 | cont1.74 |
| A12 | *N. gonorrhoeae* | PID332 | ABZO01000092 | + | 6712 | 8886 | cont1.92 |
| A12 | *N. gonorrhoeae* | SK-92-1035 | ABZQ01000088 | + | 29705 | 31935 | cont1.88 |
| A12 | *N. gonorrhoeae* | SK-92-679 | ABZP01000105 | + | 6714 | 8904 | cont1.105 |
| A12 | *N. meningitidis* | FAM18 | [AM421808](http://www.ncbi.nlm.nih.gov/nuccore/AM421808) | + | 1297477 | 1299711 | NMC1310 |
| A12 | *N. meningitidis* | K1207 | ADWM01000040 | - | 13502 | 11309 | ctg00049 |
| A12 | *N. meningitidis* | S0108 | ADWN01000171 | + | 6285 | 8475 | ctg00199 |
| A12 | *N. meningitidis* | Z2491 | [AL157959](http://www.ncbi.nlm.nih.gov/nuccore/AL157959) | + | 1494377 | 1496523 | NMA1589 |
| A13 | *N. gonorrhoeae* | 1291 | ABZF01000081 | + | 6713 | 8895 | cont1.81 |
| A13 | *N. gonorrhoeae* | DGI2 | ACIG01000064 | + | 13251 | 11078 | cont1.64 |
| A13 | *N. gonorrhoeae* | DGI18 | ABZH01000053 | - | 14019 | 11840 | cont1.53 |
| A13 | *N. gonorrhoeae* | FA1090 | [AE004969](http://www.ncbi.nlm.nih.gov/nuccore/AE004969) | - | 632346 | 630076 | NGO0641 |
| A13 | *N. gonorrhoeae* | FA6140 | ABZI01000052 | - | 14058 | 11840 | cont1.52 |
| A13 | *N. gonorrhoeae* | PID24 | ABZN01000108 | - | 14017 | 11878 | cont1.108 |
| A13 | *N. gonorrhoeae* | NCCP 11945 | [CP001050](http://www.ncbi.nlm.nih.gov/nuccore/CP001050) | + | 1040315 | 1042509 | NGK_1272, NGK_1273 |
| A18 | *H. influenzae* | 6P18HI | ABWW01000001 | + | 55905 | 57931 | CGSHi6P18H1_05686, CGSHi6P18H1_05691 |
| A19 | *N. meningitidis* | 053422 | [CP000381](http://www.ncbi.nlm.nih.gov/nuccore/CP000381) | + | 1288241 | 1290185 | NMCC_1287, NMCC_1288 |
| A19 | *N. meningitidis* | 8013 | FM999788 | - | 1023645 | 1021689 | NMV_1023, NMV_1022 |
| A20 | *H. influenzae* | R3021 | AAZE01000001 | - | 208296 | 206245 | CGSHi22421_08853, CGSHi22421_08848, CGSHi22421_08843 |
